# Supplementary material for: Zoonotic hepatitis E virus spreads through environmental routes in pig herds – A phylodynamic analysis
Source: PLoS Pathog. 2025 Nov 24;21(11):e1013710. doi: 10.1371/journal.ppat.1013710 (PMC12677782; doi:10.1371/journal.ppat.1013710)
Supplement: S1 Appendix. — Text A. Farm description. Preparation of sampling kits for sample collection. Collection of boot sock and blood samples. Prevention of HEV transmission via researchers. Laboratory protocol. Criteria to define the onset of infection and infectious period on pen-level. HEV sequence protocol and alignment. Table A. Farm characteristics, production parameters and biosecurity measures. Table B. Ct-values of boot sock (BS) and fecal dropping (FD) samples of farm A, per pen, for each week of sampling. Table C. Ct-values of boot sock (BS) and fecal dropping (FD) samples of farm B, per pen, for each week of sampling. Table D. Time of onset and duration of pen-level infection per farm compartment and batch. Table E. List of HEV genotype 3c complete genome sequences retrieved from NCBI used for the phylogenetic maximum likelihood tree. Table F. Model specification and priors for the multitype birth-death models of farm A and B. Table G. Results of MTBD models of farm A and farm B. Table H. Model specification and priors for the coalescent skyline analysis of farm A. Table I. Estimated posterior evolutionary rate distributions of batch B and C sequences in the coalescent Bayesian skyline analysis. Fig A. Suggested parent strains and regions of recombination of nine recombinant sequences from farm A. Fig B. Schematical overview of the models for between-pen transmission of compartment 2 in farm B. Fig C. Sampling scheme of fecal dropping, boot sock and blood samples per farm. Fig D. Schematical overview of multitype birth-death model for farm A, batch B. Fig E. Change times and parameters separately estimated before vs. after change times. (DOCX) [file ppat.1013710.s001.docx]

Supporting information for

Zoonotic hepatitis E virus spreads through environmental routes in pig herds - a phylodynamic analysis

M. Meester ^1^, C. Valenzuela Agüí* ^2,3^, T.J. Tobias* ^1,4^, R.W. Hakze van der Honing ^5^, C. Guinat ^6^, M. Bouwknegt ^7^, L. du Plessis ^2,3^, E.A. J. Fischer ^1^, F. Harders ^5^, M. Spaninks ^1^, T. Stadler ^2,3^, W.H.M. van der Poel ^5^, J.A. Stegeman ^1^

* Equal contributions

^1^ Department of Population Health Sciences, Faculty of Veterinary Medicine, Utrecht University, Utrecht, the Netherlands;

^2^ Department of Biosystems Science and Engineering, ETH Zürich, Basel, Switzerland;

^3^ Swiss Institute of Bioinformatics, Lausanne, Switzerland;

^4^ Royal GD Deventer, the Netherlands;

^5^ Wageningen University and Research, Lelystad, the Netherlands;

^6^ IHAP, Université de Toulouse, INRAE, ENVT, Toulouse, France;

^7^ Vion Food Group Boxtel, the Netherlands

**This PDF file includes:**

Text A

Table A to I

Fig A to E

## Text A

### Farm description

Both farms consisted of farm buildings with a central corridor, and compartments, which are spaces completely separated by concrete walls. Within farm compartments, pigs are housed in small groups, ‘pens’, and adjacent pens often share a feed trough and pigs can have direct contact with each other through bars (Fig 2A). Pigs are housed in the same compartment from about 10 to 28 weeks of age. Every week, pigs from a few compartments that have reached slaughter weight are transported to slaughter, after which the compartment is cleaned, and a new batch of pigs enters. Some further characteristics and biosecurity measures of the farms are listed in Table A.

### Preparation of sampling kits for sample collection

Sampling kits were prepared for both farms each week, and entailed zipper bags per pen, with two gloves, and material for collecting a boot sock (BS) and fecal dropping (FD) samples. Stirring rods were put inside 50 mL tubes (Sarstedt, Nümbrecht, Germany) to collect FD samples. The samples were identified by unique labels containing farm ID, date, batch A/B/C, week number, compartment number, sample type, and pen number. Every three weeks two extra sampling kits (blank samples) were made per farm as negative control, and followed all procedures except sampling itself, to assess contamination of samples between preparation and laboratory processing. For both farms a weekly sampling scheme was printed to fill out the number of pigs per pen, any deviance from the research protocol during sampling, and health findings like gastro-intestinal or respiratory signs.

### Collection of boot sock and blood samples

BS samples were collected by treading through the pen while purposefully stepping into feces and urine [1]. The samples were collected in addition to FD samples because BS have a higher sensitivity to detect presence of disease in case of a low proportion of HEV infected pigs, and therewith are useful to determine the onset of infection per pen [2]. With BS samples, floor material inside pens is soaked up, so they can be PCR positive for HEV weeks after the end of shedding of pigs. Therefore, they are not useful to determine the infectious period, yet are assumed to indicate environmental persistence of HEV in pens [2]. Therefore, BS were first collected weekly until FD samples from pens were positive, to determine start of infectiousness, and secondly in the last weeks prior to slaughter, to determine environmental persistence.

Blood was collected in batches B and C on two occasions, in the first week of the batch to determine HEV serostatus of pigs entering the compartments, and in the last week prior to slaughter to verify the infection status of the pen (i.e. if BS and FD samples suggest shedding HEV, pigs must have seroconverted). A sample size calculation for ‘freedom of disease’ with imperfect tests was performed with an assumed test sensitivity and specificity of 95%, a design prevalence of 80% and a 95% confidence interval, returning a required sample size of four randomly selected pigs per pen, both for pens with 9 and with 12 pigs in total [3]. Five mL of blood was collected using Monovette serum tubes (Sarstedt, Nümbrecht, Germany) and 18G*1.5” 1.2*40 mm needles for the pigs in the first week of batch B and C and 18G*2” 1.2*50 mm needles for the last week of batch B and C (Braun, Melsungen, Germany). Pigs with a general impression of illness or in which blood collection did not succeed were excluded from blood collection. Ear tags of pigs in the first week of the batch were noted and small cuts in the ear tags were made, in order to easily identify the same pigs for sampling in the last week of the batch.

### Prevention of HEV transmission via researchers

Specific measures were taken to prevent spread of HEV particles between pens and compartments by the researchers while collecting the samples. Farm-specific overalls and boots were worn during each sampling and washed afterwards at the farm. Boxes with sampling material stayed in the central corridor of the barn, and only the prepared sampling kits per pen for the specific compartment were brought inside. Garbage bags were also used for one compartment only. New coveralls, hairnets and gloves were worn and boots cleaned per compartment. While entering a pen, new boot covers were put on, covering the farm boots, prior to any floor-contact, as well as an extra pair of gloves. BS were only put on after entering the pen and on top of boot covers, to ensure the sample only contained material of the specific pen. Only one person entered each pen to collect the samples, except during blood collection when maximally three persons entered (two for blood and one for feces collection).

### Laboratory protocol

The laboratory protocol to process FD and BS has been described in detail elsewhere [2]. In short, FD samples were suspended in tryptose phosphate buffer 2.95% with gentamycin to obtain a 10% solution, vortexed, incubated for 30 minutes (min) and vortexed again, and centrifuged for 10 min at 2,500 x *g*. BS were suspended in phosphate-buffered saline (PBS) to obtain a 20% solution, and homogenized using a Stomacher for 1 min. From the supernatant of both samples, 150 µl was transferred to a Micronics tube and 150 µL of DNA/RNA shield was added. All processed samples were stored at -80 °C for maximally one month until further usage. Subsequently, RNA was extracted using the Quick-DNA/RNA Viral magbead Kit (Zymo Research, Irvine, CA, USA). Hereafter, HEV RNA was detected by real-time RT-PCR using Taqman FastVirus 1-step master mix [4]. PBS was included as negative control and HEV genotype 3 from hepatocytes of an infected pig as positive PCR control, and the controls were included in each run. A Ct-value < 40 was deemed positive for HEV RNA.

Blood serum samples were centrifuged for 10 minutes at 1500 g, after which 1 ml of serum was transferred to Micronics tubes. Serum was stored at -80 °C before further analysis within three months after collection. HEV antibodies were detected by an HEV genotype 3 antibody ELISA [5]. The ELISA was done using an ELISA plate reader Greiner X92. A recombinant Baculovirus expression product of HEV ORF-2, genotype 3 was produced and coated onto polystyrene ELISA plates [5]. ELISA plates were coated by dispensing 100 μL diluted Baculo-protein to each well in the ELISA plate. All reagents were equilibrated to room temperature for at least half an hour. Serum was pre-diluted (1:20) and diluted again (1:5) in the ELISA plates. Two positive and two negative controls were included. MCA anti-swine IgM, anti-swine L-chain conjugate 1:8,000 and horseradish peroxidase (HRPO) conjugate 1:8,000 were diluted in block buffer and 100 μL of the diluted conjugates were dispensed to each well. After a washing step, ready-to-use 3,3’,5,5’-Tetramethylbenzidine (TMB) substrate was added to visualize the binding of HRPO to IgM and IgG antibodies. The optical density (OD) of the substrate was measured at 450 nm by an ELISA plate reader and compared to the positive control samples to calculate Percentage of Positivity (PP) values. Samples with a PP value equal to or higher than 43.5% were deemed HEV seropositive [6].

### Criteria to define the onset of infection and infectious period on pen-level

#### Onset of *infection*

- Infection of pigs in a pen was assumed to have occurred in the week that a BS sample was positive for the first time, if followed by a positive FD sample in the following week, or a positive BS sample in the following week and a positive FD sample after two weeks.
- Infection was assumed to have not yet occurred, if a single BS sample was positive in one week, while FD and BS samples were negative in that week and the week before and after.
- The onset of infection in a pen is defined as the week of a first positive sample, minus half a week to adjust for the weekly sampling interval.

#### Infectious period:

- FD sample results are considered false negative, if the sample results of the week before and after the sample were positive.
- FD sample results are considered false positive, if the FD sample results of three or more weeks prior to it were negative.
- For three pens, two consecutive FD samples were negative, while before and after those weeks, samples were HEV positive. In those cases, a decision about true or false negative was based on the Ct-values of the other samples. E.g. if the sample before the two negative weeks had a Ct-value of 32, and the sample after the two negative weeks a Ct-value of 39.5, the sample with Ct-value of 39.5 is considered false positive, thus HEV negative and those three weeks were not considered for the infectious period.
- The duration of the infectious period per pen was defined as the number of weeks from the first positive BS sample to the last positive FD sample per batch, plus two times half a week to adjust for the weekly sampling interval.

### HEV sequence protocol and alignment

#### Sample preparation, RNA isolation and pre-enrichment

A pre-enrichment procedure was required before the RNA isolation, during which 500 µL supernatant of the FD or BS sample was filtrated with a 0.45µm filter (Millipore, ref. UFC30HV00) to purify the sample of most bacteria and other contaminants. Thereafter 1% complete EDTA-free protease inhibitor cocktail (Roche, ref: 11873580001) was added to the sample to protect the target virus during the following incubation step. A benzonase treatment at 37°C for 4 hours was performed (0.25µL benzonase (250 U/µl, Roche) and 0.5µL 2mM MgCl2 was added to 500µL of sample) to remove free (non-encapsulated) nucleic acids and enrich the sample for the HEV target. Trizol was added to the benzonase treated sample and RNA was isolated with the Direct-zol RNA isolation Kit (Zymo Research). To increase the amount of HEV, a pre-enrichment was performed with random primers, in a so called Sequence-independent Single-Primer Amplification (SISPA enrichment) [7]. Amplified DNA was cleaned using AMPure XP beads (Beckman Coulter) at a 0.8x ratio, programmed on a Biomek 4000 [8, 9]. Quality control was performed using automated gel-electrophoresis using a HSD5000 tape (Tapestation, Agilent) and dsDNA was measured using Quant-it Technology (ThermoFisher) on ClarioSTAR (BMG Labtech) both as per as manufacturer’s instructions [10]. In addition, detection, and quantification of the enriched HEV RNA was undertaken by HEV real-time PCR as previously described [4].

#### HEV sequencing

The samples were sequenced according to the protocol in Hakze – van der Honing et al. 2025 (submitted). In short, shotgun sequence libraries were created from the pre-enriched samples using SureSelectXT HS Target Enrichment System for Illumina Multiplexed Sequencing platforms (Agilent Technologies) as per the manufacturer’s recommendations. An in-house developed HEV-specific probeset, based on 20788 HEV sequences from NCBI which resulted in a set of 23102 probes of 120 bp long, and the Agilent enrichment procedure were used. Afterwards, samples were put through quality control by analyzing the DNA profile using automated gel-electrophoresis on TapeStation 2200 (Agilent), and dsDNA concentrations were established by a fluorescence measurement using ClarioSTAR (BMG Labtech), both as per manufacturer’s instructions. The samples were multiplexed and subsequently sequenced using Illumina short-read sequencing (MiSeq). The unpolished sequence data were trimmed for adapter and read quality using BBtools (version 39.01, <https://jgi.doe.gov/data-and-tools/software-tools/bbtools/> ) with a phred score above 20. The polished reads were aligned to the closest reference using BBtools and SAMtools (version: 1.16.1). Consensus sequences were constructed after several iterations of aligning polished reads using reference-based mapping and followed by polishing the draft genome with pilon (version: 1.24). Finally, the polished reads were aligned to the final draft genome for manual inspection using the Integrated Genomics Viewer (IGV version: 2.12.3). All scripts were designed inhouse using a combination of BBtools and samtools (v1.19, <https://www.htslib.org/> ). Reading depth varied along the whole genome, with ORF2 often having a 10-100x higher depth than ORF1.

## Tables

### Table A. Farm characteristics, production parameters and biosecurity measures

| **Criterium** | **Farm A** | **Farm B** |
| --- | --- | --- |
| Number of pigs | 5,600 | 2,900 |
| Number of barns | 2 | 2 |
| Number of pigs per pen and pens per compartment | 12 pigs/pen – 12 pens/compartment | 9 pigs/pen – 12 pens/compartment |
| Outdoor access | No | No |
| Average growth rate of pigs during grower-finishing period | Not provided | 880 grams/day |
| Energy corrected feed conversion ratio | Not provided | 2.9 |
| Other farm animals on farm | No | No |
| Origin of pigs | Reared at farrow-to-finish farm from same owner | Purchased from closed farrow-to-weaning farm with different owner |
| Frequency of pigs entering / leaving farm | Weekly | Biweekly |
| Relocation of pigs within farm | Sometimes one movement from a large pen (50 pigs) to 12 pigs / pen because of lack of space | No movement during growing-finishing phase |
| All pigs enter compartment simultaneously | Yes | Yes |
| Cleaning and disinfection of pens | Cold water, high pressure cleaning | Soaking with soap – cold water, high pressure cleaning - disinfection |
| Cleaning of fomites | Infrequently | After every contact with pigs |

### Table B. Ct-values of boot sock (BS) and fecal dropping (FD) samples of farm A, per pen, for each week of sampling

Each row represents a pen, white cells represent weeks in which no sample was collected, grey and light blue cells represent weeks with HEV negative results.

### Table C. Ct-values of boot sock (BS) and fecal dropping (FD) samples of farm B, per pen, for each week of sampling

Each row represents a pen, white cells represent weeks in which no sample was collected, grey and light blue cells represent weeks with HEV negative results.

### Table D. Time of onset and duration of pen-level infection per farm compartment and batch

IQR: Interquartile range; Comp: compartment.*The infectious period of farm B is underestimated due to the timing of slaughter prior to the end of the shedding period.

| **Farm** | **Compartment** | **Duration batch B (weeks)**  **mean – median (IQR)** | **Duration batch C (weeks)**  **mean – median (IQR)** |
| --- | --- | --- | --- |
| Farm A | Comp1 | 10.7 – 11.5 (8.0 – 12.0) | 7.8 – 8.5 (5.0 – 9.0) |
|  | Comp2 | 9.2 – 9.0 (6.8 – 11.0) | 7.4 – 7.0 (6.0 – 9.0) |
|  | Comp3 | 6.4 – 6.0 (5.0 – 7.0) | 5.6 – 5.5 (4.8 – 6.0) |
| Farm B | Comp2 | 5.2 – 5.0 (4.75 – 6.25)* | -- |
|  |  | **Time of onset of infection batch B (weeks)**  **mean – median (IQR)** | **Time of onset of infection batch C (weeks)**  **mean – median (IQR)** |
| Farm A | Comp1 | 2.8 – 2.5 (2.5 – 3.5) | 0.75 – 0.5 (0.5 – 0.5) |
|  | Comp2 | 2.6 – 2.5 (1.5 – 3.5) | 3.3 – 3.5 (3.0 – 4.5) |
|  | Comp3 | 3.9 – 4.0 (3.3 – 4.5) | 1.8 – 1.0 (0.5 – 2.8) |
| Farm B | Comp2 | 11.6 – 12.0 (11.3 – 12.8) | -- |

### Table E. List of HEV genotype 3c complete genome sequences retrieved from NCBI used for the phylogenetic maximum likelihood tree.

| **Accession Number** | **Collection Date** | **Country** | **Species** |
| --- | --- | --- | --- |
| FJ705359 | 2006 | Germany | Wild boar |
| JQ013794 | 2007 | France | Homo sapiens |
| KC618402 | 2011 | Germany | Homo sapiens |
| KT159771 | 2012-10-30 | Great Britain | Homo sapiens |
| KX172133 | 2015 | Germany | Homo sapiens |
| M-614140 | 2010 | Netherlands | Homo sapiens |
| M-614141 | 2010 | Netherlands | Homo sapiens |
| MF444115 | 2015-09-17 | France | Homo sapiens |
| MH377723 | 2011 | Sweden | Homo sapiens |
| MH377727 | 2013 | Sweden | Homo sapiens |
| MH504124 | 2015-01 | Great Britain | Homo sapiens |
| MH504125 | 2015-01 | Great Britain | Homo sapiens |
| MH504126 | 2015-01 | Great Britain | Homo sapiens |
| MH504127 | 2015-01 | Great Britain | Homo sapiens |
| MH504128 | 2015-01 | Great Britain | Homo sapiens |
| MH504129 | 2015-01 | Great Britain | Homo sapiens |
| MH504131 | NA | Great Britain | Homo sapiens |
| MH504132 | 2014-12 | Great Britain | Homo sapiens |
| MH504133 | 2014-12 | Great Britain | Homo sapiens |
| MH504134 | NA | Great Britain | Homo sapiens |
| MH504135 | 2014-12 | Great Britain | Homo sapiens |
| MH504136 | 2014-11 | Great Britain | Homo sapiens |
| MH504137 | 2014-11 | Great Britain | Homo sapiens |
| MH504138 | 2014-11 | Great Britain | Homo sapiens |
| MK089849 | 2014 | Germany | Homo sapiens |
| MT362711 | 2017-08-29 | Netherlands | Homo sapiens |
| PP982543 | 2022 | France | Wild boar |

### Table F. Model specification and priors for the multitype birth-death models of farm A and B

| **Parameter** | **Prior distribution** | **Prior settings** | **Rationale** | **Reference** | |
| --- | --- | --- | --- | --- | --- |
| **Parameters similar for models of both farms** | | | | | |
| Substitution model | HKY + Γ_4_ |  | Unequal transition/transversion rates, unequal base frequencies, rate heterogeneity among sites with four categories | [11] | |
| Between-pen R_e_ | Log normal | M 2.0  SD 1.25 | High R_e_ between individual pigs, and seroprevalence in pig farms 77%, so R0 above 1 expected | [14] | |
| Rate of becoming uninfectious per pen | Fixed value | 6.5 | Equivalent to infectious period of 8 weeks | This study | |
| Sampling proportion of sampled comps before change time 1 | Fixed value | 0 | No sequences prior to batch B | This study | |
| **Parameters farm B (1 affected compartment)** | | | | | |
| Origin farm B | Gamma | α 2.0  β 0.1 | Mean of 10 weeks, based on longitudinal data | This study | |
| Change time 1 farm B | Fixed value | 0.31 | Start date of batch B | This study | |
| Change time 2 farm B | Fixed value | 0.041 | Date at which all pens were HEV positive | This study | |
| Sampling proportion farm B comp2 | Fixed value | 0.75 | Based on longitudinal data | This study | |
| Mean clock rate  farm B | Relaxed Log normal | M 0.001  SD 1.25 | Clock rate of 0.003 in literature | [12, 13] |  |
| Standard deviation clock rate farm B | Gamma | α 0.537  β 0.328 | Allow evolutionary rate to vary across branches |  | |
| tMRCA farm B – week 2 of batch B | Laplace | µ 2022.2603 scale 0.0003 | Strong prior with most values clustered around the mean, based on first transmission event according to infection data of the farm | This study | |
| tMRCA farm B – week 8 of batch B | Laplace | µ 2022.3753 scale 0.0003 | Strong prior with most values clustered around the mean, based on first transmission event according to infection data of the farm | This study | |
| **Parameters farm A (3 affected compartments)** | | | | | |
| Between-comp R_e_ | Log normal | M 1.0  SD 1.25 | Compartments are physically separated, so between-comp R_e_ < between-pen R_e_, but could still be >1 |  | |
| Comp1/2/3 – compO R_e_ | Log normal | M 1.0  SD 1.25 | Compartments are physically separated, so R_e_ possibly <1 or >1, so between-comp R_e_ < between-pen R_e_, but could still be >1 |  | |
| Rate of becoming uninfectious compO | Log normal | M 6  SD 1.25 | Somewhat lower than pens in sampled comps as compO represents both pens and farm compartments | This study | |
| Origin farm A | Gamma | α 0.5  β 2 | Mean of 1 year, because multiple introductions in farm A, so tree origin earlier than batch B | This study | |
| Change time 1 farm A | Fixed value | 0.26 | Start date of batch B | This study | |
| Change time 2 farm A | Fixed value | 0.22 | Date at which all pens were HEV positive | This study | |
| Sampling proportion farm A comp1 | Fixed value | 0.83 | Based on longitudinal data | This study | |
| Sampling proportion farm A comp2 | Fixed value | 0.75 | Based on longitudinal data | This study | |
| Sampling proportion farm A comp3 | Fixed value | 0.33 | Based on longitudinal data | This study | |
| Sampling proportion farm A compO | Fixed value | 0.0 | No sequences from this comp | This study | |
| Clock rate farm A | Fixed mean clock – uniform prior | 0.0124 – 0.0408 | Based on upper and lower bound of 95% HPD estimates of clock rates from 2 models of farm B | This study | |
| Standard deviation clock rate farm A | Gamma | α 0.537  β 0.328 | Allow evolutionary rate to vary across branches |  | |

### Table G. Results of MTBD models of farm A and farm B

| **Parameter** | **Median (95% Highest posterior density interval)** |
| --- | --- |
| **Farm B – model with tMRCA at week 8 of batch B** |  |
| Origin | 0.15 (0.13 – 0.19) |
| Between-pen R_e_ before the change time at week 14 of batch B | 3.6 (1.3 – 6.7) |
| Between-pen R_e_ after the change time at week 14 of batch B | 0.49 (0.0080 – 2.4) |
| Clock rate (subst/site/year) | 0.030 (0.021 – 0.041) |
| **Farm B – model with tMRCA at week 2 of batch B** | |
| Origin | 0.27 (0.25 – 0.37) |
| Between-pen R_e_ before the change time at week 14 of batch B | 2.1 (0.9 – 3.8) |
| Between-pen R_e_ after the change time at week 14 of batch B | 0.48 (0.018 – 2.4) |
| Clock rate (subst/site/year) | 0.0173 (0.012 – 0.023) |
| **Farm A – model with fixed clock rate based on farm B estimates** | |
| tMRCA | 1.2 (0.52 – 2.3) |
| Origin | 1.5 (0.57 – 3.4) |
| Between-pen R_e_ in comp1/2/3 between start batch B and change time | 1.7 (0.021 – 6.3) |
| Between-pen R_e_ in comp1/2/3 after the change time | 0.18 (0.012 – 0.56) |
| Between-pen/comp R_e_ in compO before start of batch B | 1.8 (0.59 – 6.0) |
| Between-pen/comp R_e_ in compO between start of batch B and change time | 2.3 (0.92 – 3.8) |
| Between-pen/comp R_e_ in compO after change time | 2.3 (0.92 – 3.8) |
| Between-comp R_e_ between start batch B and change time | 0.16 (0.02 – 0.37) |
| Between-comp R_e_ after the change time | 0.085 (0.014 – 0.20) |
| Comp1/2/3-compO R_e_ between start batch B and change time | 0.37 (0.0046 – 2.2) |
| Comp1/2/3-compO R_e_ after the change time | 0.37 (0.0046 – 2.2) |

### Table H. Model specification and priors for the coalescent skyline analysis of farm A

| **Parameter** | **Prior distribution** | **Prior settings** | **Rationale** | **Reference** |
| --- | --- | --- | --- | --- |
| Substitution model | HKY + Γ_4_ |  | Unequal transition/transversion rates, unequal base frequencies, rate heterogeneity among sites with four categories | [11] |
| Mean of uncorrelated relaxed clock rate | Uniform prior | 0.0124 – 0.0408 | Clock rate based on farm B BD model | This study |
| Standard deviation uncorrelated relaxed clock rate | Gamma | α 0.537  β 0.328 | Allow evolutionary rate to vary across branches |  |
| Number of dimensions for population size and group size |  | 5 | One effective population size estimated per three weeks of time |  |

### Table I. Estimated posterior evolutionary rate distributions of batch B and C sequences in the coalescent Bayesian skyline analysis

| Batch | Min. clock rate (subst/site/year) | 25% quantile of clock rate (subst/site/year) | Median clock rate (subst/site/year) | 75% quantile of clock rate (subst/site/year) | Max. clock rate (subst/site/year) |
| --- | --- | --- | --- | --- | --- |
| B | 0.000311 | 0.000846 | 0.0118 | 0.0558 | 0.0918 |
| C | 0.000302 | 0.00139 | 0.0148 | 0.0465 | 0.0664 |

## Figures


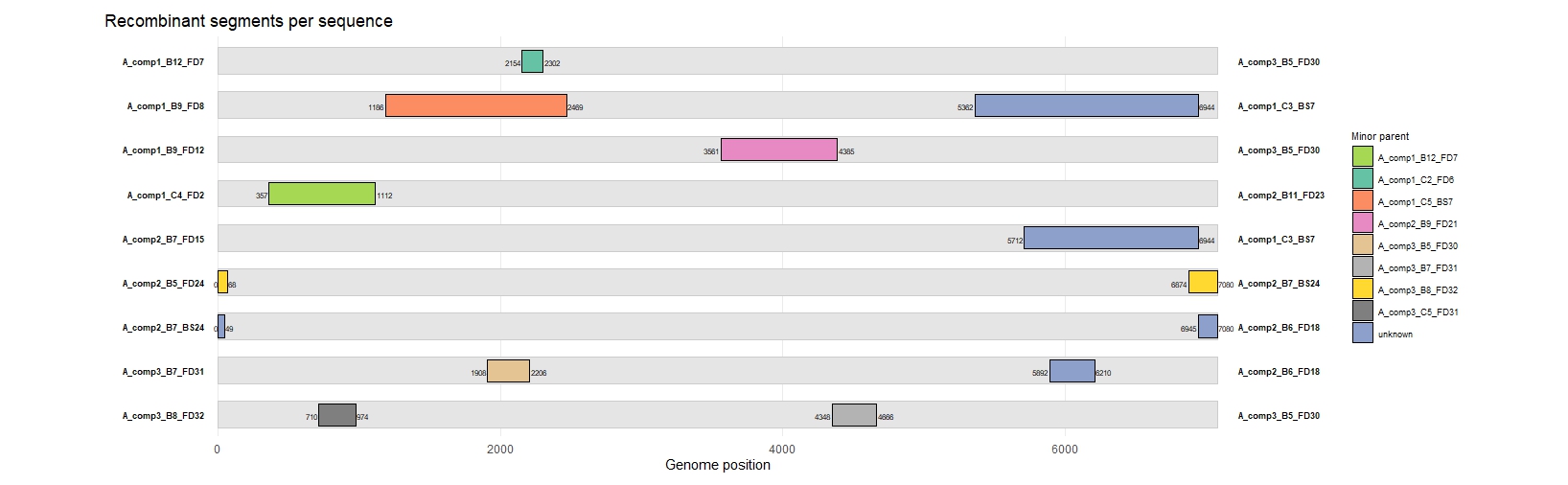


### Fig A. Suggested parent strains and regions of recombination of nine putative recombinant sequences from farm A


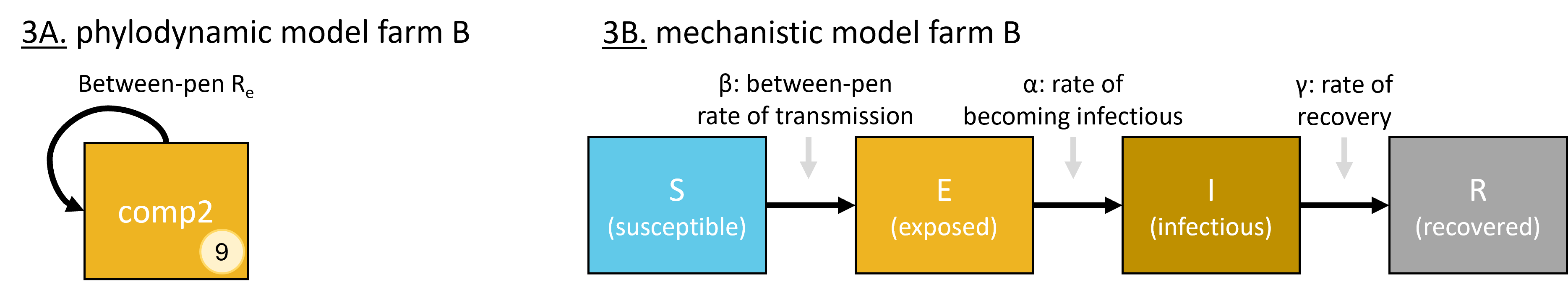


### Fig B. Schematical overview of the models for between-pen transmission of compartment 2 in farm B.

3A: Phylodynamic birth-death model for compartment 2 with number of pens from which a sequence is available in the circle. 3B: Mechanistic model for compartment 2 with the square shapes representing the number of pens in a certain state during a time interval.

### Fig C. Sampling scheme of fecal dropping, boot sock and blood samples per farm.

Legend: Colored cells represent sample types, numbers inside colored cells represent number of pens sampled per week, or for blood collection the number of animals sampled per week.


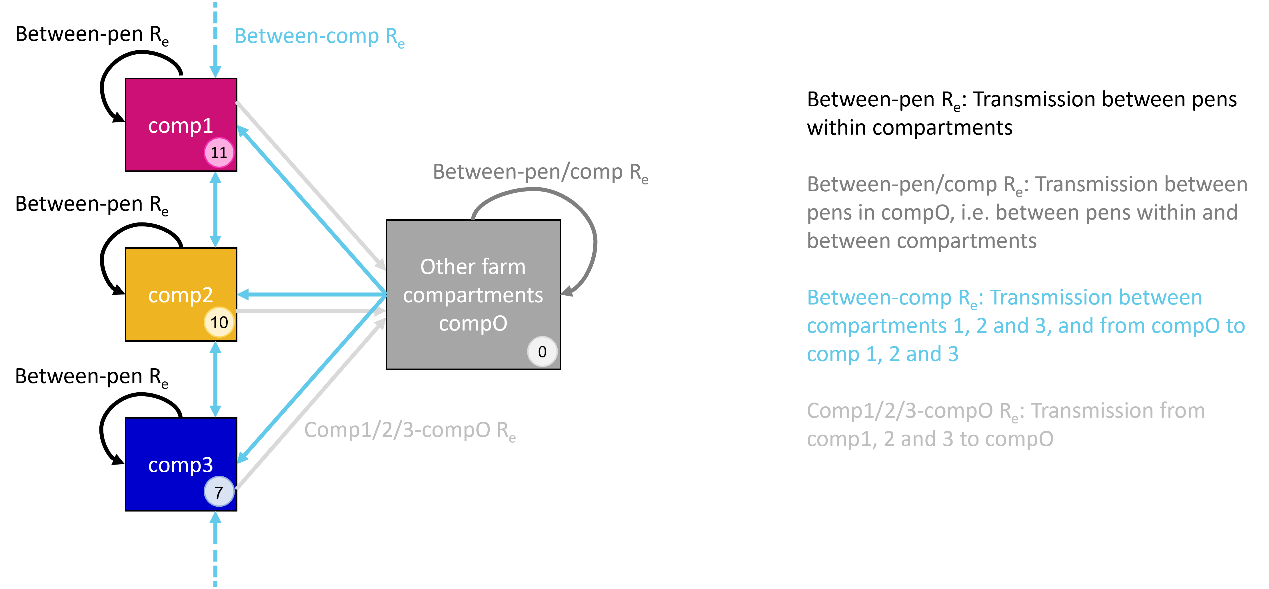


### Fig D. Schematical overview of multitype birth-death model for farm A, batch B.

Legend: Squares represent types, arrows represent estimated transmission rates as R_e_’s and encircled numbers show the number of whole genome sequences per type.


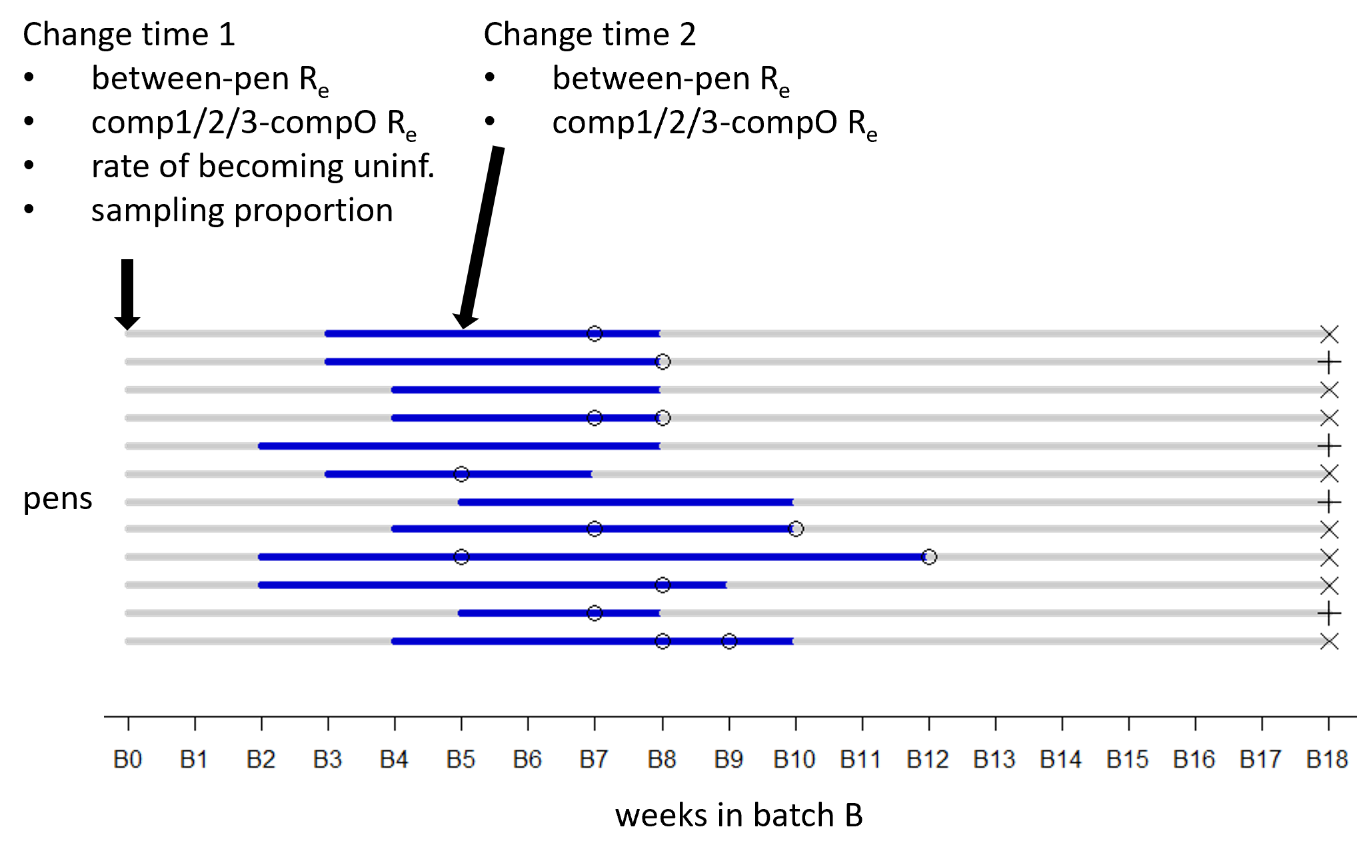


### Fig E. Change times and parameters separately estimated before vs. after change times.

# References

1. van der Wolf, P., et al., *Salmonella Typhimurium environmental reduction in a farrow-to-finish pig herd using a live attenuated Salmonella Typhimurium vaccine.* Porcine Health Manag, 2021. **7**(1): p. 43.

2. Meester, M., et al., *Evaluation of Non-Invasive Sampling Methods for Detection of Hepatitis E Virus Infected Pigs in Pens.* Microorganisms, 2023. **11**(2).

3. Cameron, A.R. and F.C. Baldock, *A new probability formula for surveys to substantiate freedom from disease.* Prev Vet Med, 1998. **34**(1): p. 1-17.

4. Jothikumar, N., et al., *A broadly reactive one-step real-time RT-PCR assay for rapid and sensitive detection of hepatitis E virus.* J Virol Methods, 2006. **131**(1): p. 65-71.

5. Van der Poel, W.H., et al., *Development and validation of a genotype 3 recombinant protein-based immunoassay for hepatitis E virus serology in swine.* Braz J Med Biol Res, 2014. **47**(4): p. 334-9.

6. Meester, M., et al., *Repeated cross-sectional sampling of pigs at slaughter indicates varying age of hepatitis E virus infection within and between pig farms.* Vet Res, 2022. **53**(1): p. 50.

7. Chen, E.C., et al., *Using a pan-viral microarray assay (Virochip) to screen clinical samples for viral pathogens.* J Vis Exp, 2011. **50.**

8. Webb, G.W. and H.R. Dalton, *Hepatitis E: an underestimated emerging threat.* Ther Adv Infect Dis, 2019. **6**: p. 2049936119837162.

9. *SureSelect XT HS Target Enrichment System*. 2022 [Accessed on 2023-11-09]; Available from: <https://www.agilent.com/cs/library/usermanuals/public/G9702-90000.pdf>.

10. thermofisher(naamwijzigen), *Comparison of accuracy and precision of Quant-iT and Qubit dsDNA quantification assays*, T.F.S. Inc., Editor. 2023.

11. Shapiro, B., A. Rambaut, and A.J. Drummond, *Choosing appropriate substitution models for the phylogenetic analysis of protein-coding sequences.* Mol Biol Evol, 2006. **23**(1): p. 7-9.

12. Purdy, M.A. and Y.E. Khudyakov, *Evolutionary history and population dynamics of hepatitis E virus.* PLoS One, 2010. **5**(12): p. e14376.

13. Nakano, T., et al., *New findings regarding the epidemic history and population dynamics of Japan-indigenous genotype 3 hepatitis E virus inferred by molecular evolution.* Liver Int, 2012. **32**(4): p. 675-88.

14. Bouwknegt, M., et al., *Estimation of hepatitis E virus transmission among pigs due to contact-exposure.* Vet Res, 2008. **39**(5): p. 40.
