# Supplementary material for: Zoonotic hepatitis E virus spreads through environmental routes in pig herds – A phylodynamic analysis
Source: PLoS Pathog. 2025 Nov 24;21(11):e1013710. doi: 10.1371/journal.ppat.1013710 (PMC12677782; doi:10.1371/journal.ppat.1013710)
Supplement: Attachment — Submitted filename: PLOS_WP4_rebuttal_2025_09_10_V2.docx [file ppat.1013710.s004.docx]

# Response to the reviewers

We thank the editor and all four reviewers for their thorough evaluation of and constructive comments on our manuscript, entitled ‘ Zoonotic hepatitis E virus spreads through environmental routes in pig herds – a phylodynamic analysis’. We have carefully revised the manuscript according to their suggestions. Below, we provide detailed responses (in blue printed text) to each comment and indicate which changes were made in the text.

**Summary**

Reviewer #1: Meester and colleagues present a comprehensive study of the transmission dynamics of the HEV on pig farms. This research is important for understanding HEV outbreak scenarios, with significant implications for improving outbreak management and overall public health. The authors used sophisticated phylogenetic models to shed light on how HEV is transmitted within farm settings, particularly between pens and compartments. The study is well written and thoroughly explained, and it is of importance for human and animal safety.

Reviewer #2: The article “Zoonotic hepatitis E virus spreads through environmental routes in pig herds – a phylodynamic analysis” by Meester et al describes a well-done epidemiological surveillance study of HEV environmental transmission in two pig farms. The authors were able to track HEV transmission and dynamics in three pig batches through phylogenetic means, and determine the effective reproduction number. The authors found differences in transmission dynamics between both farms, likely due to the biosecurity measures taken.

Reviewer #3: In this study, epidemiological field data and genetic sequencing data are integrated into phylodynamic models. The objective of this study is to quantify the contribution of environmental transmission routes to the epidemiology of HEV in agricultural holdings. This will provide a scientific basis for advising farmers and other stakeholders on effective measures to interrupt HEV transmission chains in pig production.
The present study is founded upon a comprehensive dataset, comprising weekly faecal samples from stables in several compartments on two farms over a period of eight months, coupled with whole genome sequencing of HEV. This is the first instance of phylodynamic models being employed to study the virus transmission dynamics of HEV on such a detailed scale.
This manuscript presents a clear, well-designed, and highly relevant study that makes a valuable contribution to the field. The research question is articulated with clarity, the methodology is rigorous, and the findings are presented and interpreted in a convincing and excellent manner. The work is clearly written and well structured, and will be of significant interest to the journal's readership.

Reviewer #4: This is a good study performing detailed high density sampling and sequencing, and advanced phylodynamic analysis of hepatitis E virus in two commercial pig farms in the Netherlands. The whole genome sequences in this study were collected within a year, and were used to show introductions on farms, spread within the farm and between batches of animals, and the difference that on-farm biosecurity measures can make to control the virus.

We would like to thank all reviewers for the compliments regarding the methods we undertook and the relevance of the study.

**Major issues**

Reviewer #2: The study is a well-done study with clear public health message. However, it does not seem to fit the scope of PLOS Pathogens, it rather fits more for an epidemiologic surveillance type of journal. Environmental transmission routes of HEV is not novel, even if this is the first study assessing its importance in pig farms in which pigs are in separated compartments. The study could be more concisely described, at times it is unclear what the authors mean, and it can likely be shortened, particularly the discussion.

We appreciate the reviewer’s positive assessment of the study’s quality and relevance for public health. We respectfully believe that the study does fit within the scope of PLOS pathogens, as it entails original research regarding transmission mechanisms of a virus. Specifically, the quantification of environmental transmission and the demonstration that the virus can transmit between pigs separated in space or time are novel. Regarding manuscript length, we acknowledge the comment and have carefully reviewed the text for conciseness and clarity. However, because other reviewers recommended adding explanatory details to the discussion, we have aimed to balance brevity with completeness. We hope that the current version addresses this concern adequately in light of all feedback received.

**Minor issues**

Reviewer #1

It would be beneficial for readers to know whether HEV acquired amino acid substitutions during the study. Visualising these substitutions could provide deeper insights into the virus evolutionary dynamics within the farm environment.
It would also be interesting to explore whether there are specific genomic regions that are more prone to mutations in this setup. Identifying such regions could improve our understanding of HEV adaptability and transmission mechanisms.

We agree that analysing amino acid substitutions and region-specific selection pressures would provide valuable insights into HEV evolutionary dynamics. However, these questions fall beyond the scope and objectives of the present study, which primary aimed to quantify transmission routes using combined sequence and longitudinal data. The dataset is indeed rich, and we have made all sequences publicly available to facilitate future analyses on HEV evolution, adaptability, and selection mechanisms.

The authors have discussed the role of hygiene measures in HEV transmission. However, what about viral load in the animals? Has this been quantified? And do the authors think that this could also influence transissmion dynamics?

The viral load in animals was not quantified, as we sampled at the pen level rather than from individual pigs. We assessed PCR Ct-values of the pen-level samples (also shown in supplementary files), but no consistent pattern over time could be identified. This may be due to the fact that weekly pooled samples likely contained feces from different pigs within a pen, each with varying levels of HEV excretion. We agree that transmission probabilities are likely dose-dependent and that individual variation in viral shedding could influence transmission dynamics. However, because the observational unit in our study was the pen, we expect such within-pen variability to be averaged out to a large extent in our analysis.

Reviewer #2

Some statements need to be softened, as per example HEV is not the primary cause of viral hepatitis worldwide, it is “a” main cause. Perhaps the authors meant “acute viral hepatitis”?

We thank the reviewer for this correction. The text has been revised accordingly in line 48 and 63.

- Yes, HEV has a short genome, but it is not as small as HBV, perhaps take out “small” in Line 61. Agreed.

The word ‘small’ has been removed (line 62).

- Also, please state that it 1 in 8 people having been infected is just an estimate.

Agreed. We have clarified in line 63 that this number is an estimate.

- The authors also point out that human infection of HEV is mainly caused by consumption of raw or undercooked pork, and that this route of infection can be controlled by preventing HEV spreading between pigs. I would suppose that more than the consumption of raw or undercooked meat, human infections due to contact with infected pigs at pig farms or slaughters is also high. Consumption of raw or undercooked meat can also mean that the message is to cook well the meat before consumption.

We agree that proper cooking of pork is an effective measure for preventing infection through contaminated food. However the epidemiology of HEV infection in humans remains incompletely understood. For example, HEV infections also occur among people with a vegetarian diet, suggesting that other routes such as environmental exposure or direct contact may play a role. Still, the European Food Safety Authority and other authorities still consider consumption of undercooked pork the main route of infection. We have added a sentence in line 67-69 to acknowledge additional possible routes of human infection.

- The authors could state what is the status on the development of vaccine and other treatments.

Thank you for the suggestion. A sentence was added in line 71 noting the current absence of an HEV vaccine for pigs, as vaccination could affect both virus evolution and transmission dynamics. We did not include information about human vaccines or treatments, as the specific focus of this study is on HEV transmission in pigs.

- Many major clades in the ML tree in Fig 2C has low support. Either include in your discussion or try another method (perhaps Bayesian).

Following the reviewer’s suggestion, we reanalysed the data after masking recombinant regions (see next comment). This substantially improved support values for multiple clades, as shown in the updated Figure 2C.

- Be wary on using complete genomes for your phylogenetic analyses, I would suggest to also do the analysis taking out segments with recombinant signal

We are very grateful for this comment. Based on discussions with HEV experts, we initially assumed that recombination was rare for HEV gt3 and therefore did not test for it. We have now conducted a recombination analysis and identified a significant signal for farm A, but not for farm B. We think this signal could reflect true recombination or sequence mixtures resulting from pooled samples. The analyses have been repeated after masking recombinant regions in nine sequences. The overall ML tree remained consistent, with only minor changes in sequence order within clades and overall higher support values for multiple clades. In the phylodynamic analyses this resulted in very small differences in the estimated parameter values and their HPDIs, however our conclusions remain unchanged. We have added information about the recombination analysis in the results (line 160-163), methods (line 416-423) en added fig. S1 which shows the nine farm A sequences with putative recombination regions.

- The authors state the no temporal signal was found and therefore no evolutionary rate could be detected this way. I would suggest doing a recombination analysis, and see if by removing those regions with recombinant signal, you will get temporal signal to estimate the evolutionary rate and compare to the one you inferred by using longitudinal infection data.

Thank you for this helpful suggestion. We performed the proposed recombination analysis using RDP4 and masked the identified regions of recombination. The resulting ML tree (Fig 2c) showed improved topological resolution with some additional subclades and slightly shorter branches, but no temporal signal emerged, probably because of the limited period of sampling. Line 333-336 in the discussion mention this fact.

- The figure legends lack detail and should be below the figures as instead of somewhere in the middle of the manuscript to avoid going up and down in the document.

Thank you for the comment. We have improved the figure legends and added additional details. We read that PLOS Pathogen asks to place the figure titles and legends right after first mentioning them. Yet, we have now repeated them at the bottom of the revised manuscript for convenience, and the figures are uploaded separately to make it easier to assess them.

- Please include a scale bar in all your phylogenetic trees

This scale bar has now been included.

- I suppose the number at the nodes of the trees is support value?

Yes, they represent branch support values. This is now explicitly noted that in the legends of the figures.

- The authors could consider updating their dataset from March 2023, more than two years ago.

We have updated the dataset accordingly and have found one additional HEVgt 3c sequence (PP982543), sampled in 2022 and uploaded to GenBank in 2024. It has been added to the ML tree and supplementary table.

Reviewer #3

Only minor revisions are required prior to publication, as outlined in the uploaded commented PDF file in the Reviewer Attachment (please refer to the 25 comments in the PDF file). These points do not detract from the overall quality of the manuscript, but rather serve to further enhance it.

We thank the reviewer for the constructive comments provided in the PDF. We have considered all textual comments and implemented them where appropriate. Below we respond to the content-related questions and remarks.

- Line 57 ‘our results are shared’:
  We have organized multiple outreach activities: one workshop for Dutch veterinarians and two European workshops for people working in the pig sector (part of the BiopigEE project) focused on HEV and biosecurity. Moreover, the results of the PhD project were disseminated through articles of farmer magazines and presentations to producers’ organization of pig farmers. While these details exceed the intended brevity of the Author Summary, they illustrate the impact of the study in practice.
- Line 65 ‘1:3100 blood donors’:
  We interpret the reviewer’s remark as referring to potential confusion between clinical and subclinical cases. We clarified that HEV infections among blood donors are subclinical and that these contribute to a much higher infection burden than the approximately 6,000 clinical cases. The revised sentence now emphasizes this distinction to avoid any possible misinterpretation (line 65-66).
- Line 85: Change the question to a statement.
  We acknowledge that using questions in a scientific manuscript is somewhat unconventional. However, we chose this approach intentionally to help readers, in particular those without a background in pig farming, to grasp the rational and relevance of the study.
- Line 204: Which model is better, the median Re is higher in SEIR than in the BD model:
  We appreciate this valid question. As noted in the discussion (from line 341 onwards), both models provide complementary insights. In general, integrating genetic data into transmission models can enhance inference compared to SEIR models. However, the choice of model in future applications will depend on a.o. the size of the population, availability of resources, sampling structure and prior information about strain diversity. In our case both model estimates overlap considerably suggesting similar conclusions: HEV spreads effectively in fattening pig farms (R> 1), indicating that infection will not die out without intervention.
- Fig 1: ‘What is your opinion on the matter of certain animals seroconverting despite the batch being negative for HEV (e.g. comp. 2, batch C in farm B)? This has to be discussed as well.’
  Based on the binary serological results, 14 of 144 pigs in farm B, batch C, seroconverted, suggesting exposure to HEV without onward transmission. However, inspection of the raw PP-values indicates these may represent borderline or low-level responses. Namely, the ELISA cut-off for seropositivity is 43.5 (but ‘doubtful’ between 43.5 and 53) and the PP-values of farm A ranged from 0 to 430. The average PP-value of the positively tested pigs in farm B batch C is 69, while for farm A the average is 203. If we would take 53 as cut-off, 8 out of 14 pigs from farm B batch C are no longer classified as seropositive. To reflect this nuance, we have added information on PP-value distribution in the Results (line 153-155) and discussed possible low-level cross-reactive responses in the Discussion (line 293-298).

Reviewer #4

I have a number of suggestions and comments which will hopefully improve the clarity of the work:

- Please add in the abstract / author summary and introduction the country and year where the sampled farms are located (this will greatly assist farmers veterinarians and policy makers to be able to find your study and quickly understand if its conclusions are applicable to their situation).
  We have added this information in line 26 (abstract).
- Results
  Since the Methods come at the end of the paper, please add a few more basic details into the early part of the Results section.
  Additional details about prior positivity, the independence of the farms, type of collected samples, and reference to the Supplementary materials have been added (line 115-119, line 123). Information about sequence alignment and recombination analyses has also been included for clarity (line 158-162).
- line 106 - 109. Please add the year of the sampling (2022).
  Done.
- Also, are the farms independent of each other ? e.g. they are >10km apart ?
  Yes, and clarified in line 115.
- line 139 - 140. "yielding 89 high-quality genomes", please can you indicate the length in base pairs (and/or coverage).
  We have added the coverage and alignment length in line 158-159 (results) and information about the sequence lengths in line 413 (methods).
- line 143 "all European HEV-3c WGS" - Please include the date range of the NCBI sequences in the main text as well as referring the reader to the supplementary table (e.g. 2006-2017). Also what was the length of these ? (the exact same as the new sequences, or are some just 90% of whole genome etc ?); this information can be useful for understanding how reliable the ML tree is (in general terms).
  The publicly available sequences were of length 7110 – 7340 bases. After producing a multiple sequence alignment with the newly sequenced genomes from this study, we removed all sites in the alignment with fewer than 50% unambiguous bases resulting in a final alignment length of 7080 bases. This covers >98.5% of the reference genome MT362711. The years of collection have been added to the main text in line 165.
- Line 150 - "separate clade" - presumably this has a bootstrap value of 100%, can you indicate that on the figure (there are selected bootstrap values on the figure just not in the clade you are talking about).
  The figure only shows the bootstrap support values of 70 and higher. In accordance with the findings of Hillis and Bull, 1993 (<https://doi.org/10.2307/2992540>) we take bootstrap support of >70 to roughly correspond to a probability of >95%. This is a commonly-used standard in phylogenetics.

In the newest figure, (with some strains with masked regions because of putative recombination) one can see that while the whole clade does not have bootstrap support >70, it is 83 for all but one sequence in the clade (B_comp2_B11_FD22). In the ML tree, the Farm B clade clusters together with 5 sequences from outside the Netherlands (on a long stalk), to form a clade with bootstrap support 70. Furthermore, the two clades containing Farm A sequences has bootstrap support of 76 and 100. The clade with support 76 forms a clade with bootstrap support 100 together with a sequence from outside the Netherlands (MH504125.1). Thus, it is clear that the Farm B sequences all fall into a separate clade to the Farm A sequences, and likely that they all cluster together. We have further added a scale bar underneath the tree to make clearer how different the clades are in terms of nucleotide substitutions per site.

- line 152 (figure 2) "closely related European sequences" - what are the country and year of those sequences ? impossible to tell from the figure 2C. The point being that if they are not from the Netherlands or 2022, then that is supporting the idea that these are different and independent introductions onto the farms.

A shown in the supporting table S5, there are no near-complete sequences from the Netherlands available from 2022, only two from 2010 and one from 2017. The revised ML tree now includes Genbank IDs, so that country and year can be directly cross-referenced in the Supplementary Table S5.

Figure 2C - taxa labels; for the main manuscript, I agree that you do not really want to put all the (non farm) sequence names on and need to use colours and shapes to distinguish your samples. However, the Farm A or B, and Batch A, B, C are still quite confusing. Suggest that you also put vertical bars on the figure indicating which clades are Farm A and Farm B.
The non-farm genbank IDs, and vertical bars to show which sequences are from which farms, have been added (fig 2).

Line 167 - Farm B, please add the number of sequences that went into the birth-death tree. Added to what is now line 187.

Line 232 - Comment on Figure 2C; there are only two sequences in the supplementary that don’t have dates - you might be able to run a time-scaled tree including the NCBI sequences which do have dates (and check in the records for more details than only the year)? this would establish a longer term clock rate and allow approximate estimates of the TMRCA of the independent introduction clades, prior to doing the more detailed analyses.
We agree that this would be informative. However, only three NCBI sequences have complete collection dates. Even after including these and the more specific sampling dates, we detected no temporal signal based on a strict clock (R^2 of 6*10^-2). To clarify this limitation in the data, the ‘full’ collection dates have now been added to the supplementary table S5.
Also, we would like to mention that based on the estimated clock rate in range of 10^-2^s/s/y for farm B, retrieving a clock rate with use of historical samples may not be relevant for the current research questions, as we suspect a higher evolutionary rate when sampling is performed over a shorter time interval (see the discussion). When we tried fixing the clock rate to 10^-3^s/s/y (more comparable to other literature) we obtained very distorted estimates for the timing of ancestors, all far earlier than infection according to our epidemiological data (which would imply pigs were either infected long before arriving on the farm or HEV was vertically transmitted – both of these hypotheses seem implausible because we observe a degree of clustering among farms and compartments, implying local transmission).

Figure 4A and 4B - is there overlap between the sequences in 4A and those in 4B - if so please can you indicate which ones on the figure.
Yes, 6 darkpink sequences from are included in both models, which has now been mentioned in line 493. If one may want to know exactly which ones, one can see that from the tip-labels in fig. 4A and 4B.

- Also, for Figure 4A, which is the multi-type birth death model of farm A, if you removed the bottom 4 sequences which look to be from a separate introduction, how does this affect the Re and evolutionary rate estimates ? the long connecting branches between the two clades could introduce extra uncertainty.
  Indeed it is important to assess how this affects the posterior estimates. We also applied the MTBD to sequence data only from the largest clade of the ML tree in fig. 2. That retrieved similar results. Moreover, we did an MTBD model on a different random selection of sequences (i.e. when 2 or more seqs are available from the same pen, select 1 random sequence). That approach also led to similar results.

  Discussion
  figure 2A; in Farm A you show that there are more seropositive pigs at the start of the batch than in farm B. At what age (roughly) do the pigs arrive, and is there any pig age effect in their ability to show a seropositive status if infected? perhaps include a brief statement in the discussion ?
  We agree that this may be related to age. In general, pigs are moved to fattening between 9 and 12 weeks of age. Although the exact birth-date of the pigs is unknown, we do know that farm A received piglets of lower weight than farm B, which could suggest that they were younger at arrival, and maternal antibodies can be present up to at least 9 weeks of age. We have also seen that, although some pigs of farm A were seropositive at arrival, the PP-values were almost always at least twice as high at the end of fattening, which is in support of maternal antibodies being present at arrival. A brief statement has been added (lines 293-298).
- Also in figure 4A, the TMRCAs within the upper clade are a few months prior to arrival; how do these relate to the age of the pigs?
   Some TMRCAs are even prior to birth of the pigs. Combined with the serological data at arrival and the observation that at least in batch B, shedding began only several weeks after arrival, we interpret these early TMRCAs as representing infection by a persistent ‘old strain’ rather than infection before arrival.

We hope that these revisions have addressed all concerns raised and that the revised manuscript is now suitable for publication in PLOS Pathogens.

On behalf of all authors

Marina Meester
